# Audiovestibular Manifestations in Patients with Primary Raynaud’s Phenomenon and Raynaud’s Phenomenon Secondary to Systemic Sclerosis

**DOI:** 10.3390/jcm12093232

**Published:** 2023-04-30

**Authors:** Juan Carlos Amor-Dorado, Eduardo Martín-Sanz, Virginia Franco-Gutiérrez, Ana Urruticoechea-Arana, Ana M. García-Arumí, Erwin Racines-Álava, Oscar Alemán-López, Carmen P. Simeón-Aznar, Miguel Á. González-Gay

**Affiliations:** 1 Otorhinolaryngology Division, Hospital Can Misses, 07800 Ibiza, Spain; 2 Department of Otolaryngology, University Hospital of Getafe, Carretera Toledo km 12,500, 28905 Madrid, Spain; emartinsanz@gmail.com; 3 Department of Medicine, School of Biomedical Sciences and Health, European University of Madrid, C. Tajo, s/n, 28670 Madrid, Spain; 4 Otorhinolaryngology Division, University Hospital Lucus Augusti, 27003 Lugo, Spain; virginiafrancogutierrez@gmail.com; 5 Rheumatology Division, Hospital Can Misses, 07800 Ibiza, Spain; anboliv@yahoo.es; 6 Hospital Valld’Hebron, 08035 Barcelona, Spain; 12980aga@gmail.com; 7 University Hospital of Salamanca, 37007 Salamanca, Spain; erwinracines89@gmail.com; 8 Rheumatology Division, General University Hospital of Alicante, 03203 Elche, Spain; osal.888@gmail.com; 9 Internal Medicine Service, Hospital Valld’Hebron, 08035 Barcelona, Spain; cpsimeon@vhebron.net; 10 Rheumatology Division, IIS-Fundación Jiménez Díaz, 28015 Madrid, Spain; miguelaggay@hotmail.com; 11 Department of Medicine, School of Medicine, University of Cantabria, 39011 Santander, Spain

**Keywords:** Raynaud phenomenon, audiovestibular, systemic sclerosis, balance disorder

## Abstract

Objectives: To address the prevalence of audiovestibular disorders in patients with primary Raynaud’s Phenomenon (RP). A series of patients with primary RP and secondary RP in the context of systemic sclerosis (SSc) were compared with healthy controls. Methods: A prospective multicenter observational cross-sectional study was conducted in several Otolaryngology and Rheumatology Divisions of tertiary referral hospitals, recruiting 57 patients with RP and 57 age- and gender-matched controls. Twenty patients were classified as primary RP when unrelated to any other conditions and 37 patients who met the 2013 ACR/EULAR classification criteria for SSc were classified as having secondary RP associated with SSc. Audiometric and vestibular testing (vHIT), clinical sensory integration and balance testing (CTSIB), and Computerized Dynamic Posturography (CDP) were performed. Results: As significant differences were found in the age of the two study groups, primary and secondary RP, no comparisons were made between both groups of RP but only with their control groups. No sensorineural hearing loss (SNHL) was recorded in any of our patients with primary RP and no differences were found in the voice audiometry tests with respect to controls. Four of 37 (10.8%) secondary RP patients presented SNHL. Those with SNHL were 7.03 times more likely to have a secondary RP than controls (*p* < 0.001). The audiometric curve revealed high-frequency hearing loss in 4 patients with RP secondary to SSc, and statistically significant differences were achieved when RP secondary was compared to controls in vHIT gain, caloric test, CTSIB, and CDP. Conclusions: Unlike patients with RP secondary to SSc, patients with primary RP do not show audiovestibular abnormalities. Regarding audiovestibular manifestations, primary RP can be considered a different condition than secondary RP.

## 1. Introduction

Raynaud’s phenomenon (RP) is a frequent reason for consultation in young women, consisting of vasospastic episodes triggered mainly by cold or stress, typically manifested with cyanosis or pale fingers, followed by reperfusion erythema. Its prevalence in the general population is high, about 3–5%, predominantly women (7:1). It can be classified as primary when it appears in isolation without any underlying disease (80% of cases) or secondary, when it is generally associated with an autoimmune disease, such as systemic sclerosis (scleroderma-SSc), systemic lupus erythematosus, mixed connective tissue disease and rheumatoid arthritis. The symptoms of RP are often the first sign of an underlying connective tissue disease. Patients with the secondary form are more likely to suffer more serious problems from RP, such as skin ulcers from exaggerated physiologic vasoconstriction or even gangrene from microvascular changes that cause more severe episodes, and finally trophic changes [1].

The differential diagnosis between primary and secondary RP is essential as the prognosis and treatment often differ markedly between them. Close clinical monitoring of people with primary RP is also recommended, since a secondary cause appears in up to 14.7% of cases throughout the disease, with SSc being a frequent condition in these cases [2,3,4,5].

The diagnosis of RP is clinical, but additional tests (autoimmunity and periungual capillaroscopy) are performed to help identify an underlying cause [1]. Regarding this, antinuclear antibodies (ANA) are positive in a high percentage of subjects with SSc and mixed connective tissue disease (MCTD), the entities more commonly associated with RP. The combination of both an abnormal capillaroscopic pattern and positive ANAs identifies a group of people with a high clinical probability of developing a systemic autoimmune disease. On the other hand, the normality of the capillaroscopic study and the negativity of ANA in a subject with RP indicate that the probability of developing a systemic autoimmune disease is low [5]. To date, 4 studies describe audiovestibular disorders in patients with SSc [6,7,8,9] suggesting an ischemic etiopathogenesis [9]. However, to the best of our knowledge, there is no information on auditory or vestibular disorders in patients with primary RP. The objective of the study is to determine if patients with primary RP may also present audiovestibular alterations, as occurs in RP secondary to SSc, or if the presence of a primary RP is not associated with audiovestibular damage. To address this issue, we studied a series of patients with RP both primary RP and secondary to SSc. Comparisons of patients with primary RP and RP in the SSc setting were made with healthy controls.

## 2. Materials and Methods

### 2.1. Design and Recruitment of Patients

A multicenter cross-sectional observational study was designed. As previously performed by our group to identify SSc patients [10], we used the presence of RP as the entry point for potential identification. RP was considered present if a patient had a history of any 2 of the following 3 manifestations: pallor, cyanosis, and suffusion. In all cases RP had to be observed or provoked by an expert clinician who was familiar with SSc [10]. Consecutive patients with primary RP or RP secondary to SSc, seen in the Rheumatology outpatient clinics of tertiary referral centers between March 2019 and February 2021, were evaluated for audiovestibular studies. Data from these patients were compared with gender and age-matched controls from involved hospitals. The study was approved by the Ethics Committee of Cantabria University (approval code: 2017.150).

### 2.2. Inclusion Criteria

Patients with primary RP were defined when this condition was not related to any condition, including autoimmune diseases. Patients with RP associated with SSc fulfilled the 2013 ACR/EULAR classification criteria for SSc [11].

### 2.3. Controls

Healthy volunteers from all hospitals matched for age ±5 years, sex, and ethnicity with no family history of any connective tissue disease were studied.

### 2.4. Exclusion Criteria

To determine the prevalence of audiovestibular manifestations concerning RP, all patients, and controls were asked about any history of previous audiovestibular abnormalities, head trauma and exposure to noise, ear infection, metabolic disease, renal failure, use of ototoxic drugs, and family history of hearing impairment. To avoid bias in hearing thresholds measured from patients with RP, all those who presented a previous history of middle ear dysfunction and abnormal immittance results were excluded. In addition, only patients under 45 years of age were included to avoid the possible effect of aging in the audiovestibular tests.

### 2.5. Clinical Data

Subjective hearing loss, tinnitus, vertigo, dizziness, and disequilibrium were assessed, based on definitions previously described in another study [9].

#### 2.5.1. Quantitative Hearing Loss Was Evaluated by Audiometric Tests

(1) Pure-tone audiometry: Behavioral pure-tone threshold testing, or bone and air-conducted signals were performed. Handicap impairment was calculated for the four pure-tone average (PTA) arithmetic means of 0.5, 1, 2 and 4 kHz used in the Academy formula [12,13]. Data were expressed as decibels of hearing level (dB HL). Pure-tone air and bone conduction thresholds were obtained in a sound isolation chamber with a clinical computer audiometer (Interacoustics, Model AC5, Assens, Denmark). 

Hearing loss (hypoacusis) was considered present when the audiometric tests disclosed pure-tone thresholds equal to or greater than 30 dB HL of PTA. A hearing loss difference greater than 15 dB HL between each ear in at least one frequency (0.5–4 kHz) was rated as asymmetrical.

(2) Speech reception threshold (SRT): The lowest intensity level expressed in dB HL at which the patient could correctly identify 50% of common two-syllable Spanish words from a phonetically balanced list was tested [14]. The correlation between SRT and PTA was also studied. It was considered abnormal if the differences between SRT and PTA were greater than 5 dB HL. 

(3) Free-Field audiometric speech discrimination test (SDT): It was considered abnormal if individuals were unable to identify at least 80% of common two-syllable Spanish words from a phonetically balanced list of two-syllable words [14]. In addition, the intensity in dB HL in each ear and each patient and control to achieve 100% or maximal individual intelligibility of the phonetically balanced word list and recruitment was also assessed.

#### 2.5.2. Immittance Study

(1) Tympanometry: The tympanometry scale was measured in decapascal (daPa). Static admittance (or compliance) and peak pressure were also measured in cm^3^. Peaks under 0.1 cm^3^ (a reduced peak height or a flattened curve) and over 1.5 cm^3^ were considered abnormal. In this study, pressures less than −125 or greater than 100 daPa and a flat curve were considered abnormal. Tympanogram tracings were performed using MAICO MI 24/26 Tympanometer/Pure Tone Screener, Assens, Denmark, and grouped according to the classification provided by Margolis et al. [15]. The immittance study evaluated the presence of abnormalities in the compliance of the tympanic membrane and the middle ear. The presence of a previous history of middle ear dysfunction like otitis media with effusion associated with abnormal immittance results was considered a bias in the audiometric results and therefore excluded.

(2) The stapedius reflex: Ipsilateral reflexes were elicited at 500, 1000, and 2000 Hz using 105 dB HL and at 4000 Hz using 100 dB HL (MAICO MI 24/26 Tympanometer/Pure Tone Screener, Assens, Denmark). The amplitude of the reflex, latency and timing (sustained or rapidly decaying) was quantified (reflex decay). The absence of reflex, latencies inferior to 40 or greater than 180 milliseconds, or the presence of decay in any ear were considered abnormal.

Qualitative and quantitative vestibular functional tests: All studies were performed using videonistagmoscope Ulmer VNG, Version 3.3; SYNAPSIS, Marseille, France.

(1) Spontaneous nystagmus: eye movements were recorded for at least 20 s with and without visual fixation by videonistagmoscopy registration [16]. 

(2) Gaze-evoked nystagmus was studied by videonystagmoscope as Shepard NT et al. previously described [17].

(3) Oculocephalic response (OCR) also called the “head thrust test” or Halmagyi test is performed as described by Harvey and Wood [18]. An abnormal response was recorded when the eyes drifted in the same direction as the head and clinically evident compensatory refixation saccades were necessary to reset the gaze on the stationary target. 

(4) Positional nystagmus: The presence of positional nystagmus in any direction in at least one of the four positions (supine lying, right lateral lying, left lateral lying, and head hanging position) was considered abnormal [19].

(5) Positioning test was performed according to Barany Society criteria [20].

(6) Video Head Impulse Test (vHIT). Horizontal semicircular canal function was assessed by using horizontal video-HIT (OtosuiteV®, GN Otometrics, Denmark) and following the instructions of MacDougal et al. [21].

(7) Head-Shakingtest was considered positive, when jerk nystagmus in one direction appeared [22].

(8) Vestibular responses were obtained using conventional bithermal caloric testing (30.5 °C and 43.5 °C) [23]. A video-based system was used for the acquisition and analysis of the eye response. The maximum velocity of the slow phase components of nystagmus evoked in each ear was analyzed to identify unilateral weakness and directional preponderance as determined by Jongkees’ formula. We considered a canal paresis higher than 25% as pathological. 

Positional testing, Dix-Hallpike, Head Shaking Nystagmus, and caloric tests eye movements were registered using videonystagmoscopy (Ulmer VNG, Version 3.3; SYNAPSIS, Marseille, France) with and without ocular fixation. Despite the clinical value of the Vestibular evoked myogenic potentials (VEMP), they were not included in the study since some of the centers involved did not have available the same equipment for this study.

#### 2.5.3. Balance Study 

(1) Clinical test of sensory integration and balance (CTSIB) as described Shumway-Cook A, Horak FB [24]. The examiner used the first condition (condition 1) as a baseline for comparing sway under the other 3 conditions. In conditions 3 and 4 normal adults sway 40% more than they do in condition 1. For this reason, for each specific condition, individuals were considered to have abnormal CTSIB when they were not able to maintain the position for more than 50% of the time [25]. To quantify sway, a stopwatch was used to record the amount of time the patient maintains erect standing without excessive swaying in each condition.

Patterns of postural disorientation were classified as “visually dependent” when conditions 2 and 4 fulfilled the criteria of abnormality; “surface dependent” if conditions 3 and 4 were abnormal; “vestibular loss” when an abnormality was found in condition 4.

(2) Computerized Dynamic Posturography (CDP): Standard sensorial organization test (SOT) protocol using NeuroCom SMART Equitest system, version 8.4.0 (NeuroCom, A Division of Natus, Clackamas, OR, USA) was performed. The CDP assesses both the balance system as a whole (composite) and its components, i.e., the vestibular, visual, and somatosensorial systems, in their own right [24]. If patients showed a composite less than 70% an abnormal CDP was considered. 

A magnetic resonance imaging (MRI) of the central nervous system (CNS) was done on any individual with persistent nystagmus observed when the ocular fixation test was performed or in case of any suspicion of central vestibular disorder.

### 2.6. Data Collection

Demographic and clinical features, erythrocyte sedimentation rate (ESR), and C-reactive protein (CRP) were assessed.

### 2.7. Audiologic and Vestibular Assessment: Protocol

Patients and controls were asked for the presence of hearing loss, vertigo, tinnitus, dizziness, or disequilibrium symptoms at the time of the study. All patients and controls underwent a complete ear, nose, and throat examination and the following audiological tests: pure-tone audiometric test, both aerial and bone conduction stimulus, and SRT and SDT. Impedanciometry was also performed. An MRI of the posterior fossa and brainstem was performed if the subjects had asymmetric sensory-neural hearing loss.

In all patients and controls spontaneous nystagmus, gazed evoked nystagmus, head thrust test, head-shaking nystagmus, and positional nystagmus were performed in this order. Then, the Dix-Hallpike test was done. Later, a static postural evaluation of four conditions with CTSIB was conducted and then CDP was completed. Finally, a bithermal water caloric test was also performed when was available.

### 2.8. Statistical Analysis

Continuous data were expressed as mean ± SD and categorical variables as percentages. Cases and controls were matched by age and sex and paired two groups’ comparisons (Controls vs. primary RP and Controls vs. secondary RP) were carried out via Paired-Samples *t*-Test for continuous variables or exact McNemar’s Test for categorical ones. The potential associations between auditory and vestibular results have been assessed through cross-tab generation between two variables (binary or categorical variables) and the Chi-square test. Categorical variables between both age and sex-matched groups were summarized by counts and frequencies and compared using an odds ratio (OR) with 95% CIs. 

Two-sided *p*-values less than 0.05 were considered statistically significant. Analyses were performed with the package Stata 16/SE (Stata Corp, College Station, TX, USA).

## 3. Results

Two patients with primary RP and 4 patients with secondary RP who had a history of middle ear dysfunction and abnormal tympanograms were withdrawn from the study. Fifty-seven patients with RP (20 with primary RP and 37 with RP secondary to SSc) and 57 matched controls were finally included.

### 3.1. Main Clinical Features of RP Patients

Patients with primary RP were younger than those with RP secondary to SSc (*p* < 0.001). The main epidemiological and clinical features of this series of patients are shown in Table 1. Most patients were women (96%). The mean age at the time of the study was 26.01 ± 1.15 years in primary RP and 34.43 ± 1.01 in secondary RP (*p* < 0.001). Patients with secondary RP showed a longer duration of RP (*p* = 0.01). Calcinosis, esophageal dysmotility, and sclerodactyly were only observed in secondary RP as they occurred in the setting of SSc (Table 1). It was also the case for ANA which was positive in 82% of the patients with RP secondary to SSc (*p* < 0.001). Elevation of CRP was observed in 3 (15%) of primary RP and 6 (30%) of secondary RP patients.

### 3.2. Audiovestibular Symptoms and Auditory Differences between RP Patients and Controls

#### Comparison between Patients with Primary RP and Controls 

Comparisons between patients with primary RP and controls showed no significant differences in the presence of auditory or vestibular symptoms or in hearing thresholds measured by air and bone conduction on liminal tone audiometry. No sensorineural hearing loss (SNHL) was registered in any of our primary RP patients. In addition, no differences were found in the vocal audiometry tests, neither in SRT nor in SDT results when patients with primary RP were compared with their age- and sex-matched control group (Table 2).

### 3.3. Comparison between Patients with RP Secondary to SSc and Controls 

Four of 37 (10.8%) patients with RP secondary to SSc complained of subjective hearing loss. These 4 patients were also found to have abnormal hearing loss in the audiogram. These 4 patients with secondary RP who presented with sensorineural hearing loss had positive ANA and 3 of them presented abnormalities on capillaroscopy such as giant capillaries, hemorrhages and avascular areas and neoangiogenesis.

In contrast, only one individual from the control group (a 40-year-old woman) had abnormal hearing loss in the audiogram (Table 3 and Table 4). This patient had no symptomatic hearing loss and no prior or family history of hearing loss. 

Dizziness and vertigo were the most common symptoms reported by patients with secondary RP (5 [14.7%] and 4 of 37 patients (11.8%), respectively]). With respect to this, vertigo and dizziness yielded statistically significant differences between patients with secondary RP and controls (*p* < 0.001).

Four of 37 (10.8%) patients with secondary RP had SNHL. The presence of SNHL was associated with a 7.03 times greater probability of having secondary RP than controls. (*p* < 0.001).

None of the patients showed mixed or conductive hearing loss. However, the audiometric curve revealed a high-frequency hearing loss in 4 patients with RP secondary to SSc.

Hearing thresholds data of pure-tone air and bone audiometry test in 0.5-1-2 and 4 kHz from primary and secondary RP patients are shown (Table 5).

### 3.4. Vestibular and Postural Differences between Patients with RP and Controls

No statistically significant differences were found regarding the presence of spontaneous or evoked nystagmus, abnormal oculocephalic response, Dix-Hallpike test, positional and head-shaking nystagmus test between patients with primary RP and controls (Table 6). However, unlike the primary RP patients or controls, 5 of 27 (18.5%) patients with secondary RP who underwent caloric testing had a significantly lower caloric result (*p* < 0.001) (Table 7).

Regarding vHIT results, patients with RP secondary to SSc showed significant differences when compared with controls. Patients with RP secondary to SSc exhibited significantly lower horizontal canal gains, either on the right or left side, than controls (*p* = 0.029 and *p* = 0.013, respectively). Saccades were only present in patients with secondary RP (*p* = 0.07) (Table 6).

Patients with at least one abnormal vestibular test had 3.03 times higher odds of having a secondary RP than controls (*p* < 0.001).

Abnormal CTSIB was seen more frequently in patients with secondary RP than in controls. Nine of 37 (24.3%) patients with secondary RP had an abnormal CTSIB compared with 2.7% (1/37) of controls. In this regard, statistically significant differences were achieved when secondary RP was compared with controls (*p* < 0.001). Among the CTSIB patterns, the vestibular loss was only found in patients with secondary RP 6/37 (16.2%) versus 0/37 in controls. 

Regarding CDP, 9 of 37 patients with secondary RP had an abnormal test and significant differences were found when these patients with secondary RP were compared with controls (*p* < 0.001). A pattern of vestibular loss in CDP was only present in 5 patients with RP secondary to SSc, a higher proportion than controls, although without reaching statistically significant differences (*p* = 0.08) (Table 6). These 9 of the 37 patients with secondary RP who presented with vestibular alterations had positive ANA and 7 of them presented abnormalities on capillaroscopy such as giant capillaries, hemorrhages and avascular areas and neoangiogenesis.

At the same time mild hearing loss and vestibular disorders were found in 3 patients with secondary RP. No association was found between abnormal audiovestibular symptoms, audiometric tests, abnormal vestibular tests, CTSIB, and CDP with demographic and clinical features (data not shown).

Patients with either an abnormal CTSIB or CDP had 18.6 times higher odds of having a secondary RP (*p* = 0.01) compared to controls.

## 4. Discussion

The present study constitutes the first attempt to investigate the audiovestibular manifestations in a series of patients with primary RP. Our results support the presence of auditory and vestibular dysfunction only in patients with RP secondary to SSc but not in patients with primary (idiopathic) RP. In this regard, hearing impairment was mainly registered in secondary RP (17%). Of note, most secondary RP patients presented a mild symmetrical SNHL with a flattened curve in the audiogram. Regarding this, SRT was in accordance with PTA results (within a range of difference between SRT and PTA≤ 5 dB HL). Indeed, a significant difference between the 2 thresholds (SRT and PTA) would have raised doubts about the validity of the pure-tone thresholds. Furthermore, SDT yielded good discrimination scores and a correlation between the type and degree of hearing loss, suggesting the presence of a cochlear impairment [8]. Tosti et al. [26] assessed 22 women with SSc and observed hypoacusis in 59% of them. Berrettini et al. [6] described audiovestibular symptoms in 62% of 37 patients with SSc. Furthermore, audiovestibular studies disclosed abnormalities in 41% of them. Fourteen (38%) had hearing loss (10 SNHL and 4 mixed) and 4 (11%) showed abnormal vestibular tests. Kastanioudakis et al. [27] reported the presence of SNHL in 20% of the patients with SSc. SNHL, vestibular, and balance dysfunctions were also found in a series of 35 patients with limited SSc and CENP-B antibodies [8]. These results were confirmed in other studies on both limited and diffuse SSc [6,7,8,9]. 

A potential strength of our study was the inclusion age which reduced the effect of aging. To our knowledge, a group of patients younger than 45 years with RP secondary to SSc has never been studied before. Therefore, and based on our results, we can demonstrate that audiovestibular damage in patients with SSc occurs in early stages of the disease. In contrast, the majority of patients with primary RP did not have audiovestibular abnormalities.

As previously reported, we disclosed SNHL in patients with RP secondary to SSc [8,9]. However, the frequency of hearing abnormalities in our series of patients with SSc was lower than that reported in previous studies. As discussed above, a plausible explanation for this may be that the SSc patients included in the present study were relatively young. However, our restrictive inclusion criteria helped us confirm that hearing loss in patients with SSc is probably not related to aging, exposure to ototoxic drugs, or noise. It also indicates that, unlike those with RP secondary to autoimmune diseases, most individuals with primary RP do not have hearing loss on hearing tests.

A remarkable finding observed in our study was the presence of dysfunction in vestibular and balance tests in individuals with RP secondary to SSc. Regarding this, we observed nystagmus in positional tests, reduced gain in vHIT, hypofunction in caloric test, and abnormal CTSIB and CDP test results. Dizziness was a symptom of greatest concern in patients with RP secondary to SSc. In contrast, it should be noted that patients with primary RP did not complain of vestibular symptoms. Although some of them presented abnormal CTSIB in the results of the balance study this finding was not statistically significant when compared with controls. Nevertheless, abnormal CTSIB was more evident in RP secondary to SSc.

We also found a markedly increased risk of having a secondary RP compared to controls in those patients who had an abnormal CTSIB/CDP or an abnormal vestibular test, respectively. However, we did not observe the influence of age at diagnosis or duration of RP on the development of audiovestibular disorders. We believe that this finding in relation to the analysis of vestibular function and postural control may be a valuable complement to the study of patients with SSc.

One of the limitations of the study was a relatively small sample of subjects with primary RP. In addition, information on some more vestibular studies, such as vestibular evoked myogenic potentials, could have been described, but they were not available in all centers that agreed to participate in the study. 

Despite the relatively small sample size, PTA audiometry and vHIT results on the vestibular test battery confirmed significant differences between patients with secondary RP and matched controls. In contrast, unlike RP patients in the SSc setting, those with primary RP were similar to healthy controls in terms of audiovestibular manifestations.

An issue that can be debated is the indication for performing an audiovestibular study in a patient with RP. In this sense, in the multicenter longitudinal registry study on the progression of patients with Raynaud’s phenomenon to systemic sclerosis (Very Early Diagnosis of Systemic Sclerosis Registry Study [VEDOSS]), the absence of baseline ANA was the most significant factor strongly associated with lack of progression within 5 years, with only about 11% of ANA-negative RP patients progressing to SSc [28]. For this reason, we believe that an audiovestibular study should be restricted to ANA-negative patients with RP and a high suspicion of progression to SSc, for example due to the presence of an abnormal capillaroscopy. However, further studies with larger series of individuals are needed to confirm this assumption.

In conclusion, we disclosed audiovestibular abnormalities in patients with RP. However, these abnormalities were only found in the setting of RP secondary to SSc but not in individuals with primary RP. These findings support the benign nature of the primary RP when compared with that associated with connective tissue diseases. It is possible that the presence of underlying vascular damage, even in the early stages of the disease, may be responsible for the presence of audiovestibular manifestation in patients with RP secondary to SSc.

## Figures and Tables

**Table 1:** Clinical features of 57 patients with Raynaud Phenomenon (RP).

Variable	Primary RP	Secondary RP	*p*-Value
	(n = 20) (%) *	(n = 37) (%) *	
Age (years ± SD) (range) years	/	/	/
at the time of study	26.1 ± 5.1	34.5 ± 6.9	<0.001
at the time of diagnosis of RP	19.6 ± 5.2	24.4 ± 8.8	0.03
delay to the diagnosis of scleroderma (months)	/	27.8 ± 7.9	/
duration of RP (months)	73.0 ± 42.9	116.6 ± 77.1	0.02
Sex female/male	20 / 0	35 / 0	0.27
Calcinosis	0 (0%)	6 (16.2%)	0.05
Esophageal dysmotility	0 (0%)	16 (43.2%)	<0.001
Sclerodactyly	0 (0%)	17 (45.9%)	<0.001
Digital ulcers	0 (0%)	5 (13.5%)	0.07
Abnormal capillaroscopy	0 (0%)	31 (83.8%)	<0.001
ANA †	0 (0%)	31 (83.8%)	<0.001
C-reactive protein > 3 mg/L	3 (15%)	6 (16.2%)	0.8

* Number in parenthesis indicates the total proportion of patients with a particular variable. † Antinuclear Antibodies.

**Table 2:** Epidemiological and auditory differences between controls and patients with primary Raynaud Phenomenon (RP).

Variable	Controls	Primary RP	*p*-Value
	(n = 20) (%)	(n = 20) (%)	
Sex (men/women)	0/20	0/20	
Age at the time of the study (years ± SD)	25.8 ± 5.1	26.0 ± 5.1	0.163
Individuals with abnormal audiovestibular symptoms			
Hearing loss	1 (1.7)	0 (0)	0.159
Vertigo	0 (0)	0 (0)	/
Dizziness	0 (0)	0 (0)	/
Disequilibrium	0 (0)	0 (0)	/
Individuals with abnormal hearing loss in the audiogram	1 (1.7)	0 (0)	0.159
Pure-tone-average (PTA) of air conducted signals in decibels hearing level (dB HL) ‡			
Right ear	8.15 ± 0.31	7.18 ± 0.25	0.076
Left ear	7.43 ± 0.23	6.81 ± 0.29	0.146
Pure-tone-average (PTA) of bone-conducted signals in decibels hearing level (dB HL) ‡			
Right ear	8.22 ± 0.32	7.43 ± 0.30	0.175
Left ear	7.27 ± 0.24	6.93 ± 0.24	0.449
Absence of stapedial reflex SRT in dB HL §	0 (0)	0 (0)	
Right ear	9.0 ± 5.0	8.7 ± 3.9	0.858
Left ear	10.5 ± 5.3	9.2 ± 3.3	0.33
Abnormal SRT and PTA correlation	/	0 (0)	/
Abnormal SDT ^+^	/	0 (0)	/
SDT in decibels hearing level (dB HL)			
Right ear	25.2 ± 5.7	21.0 ± 7.0	0.096
Left ear	24.7 ± 4.1	21.2 ± 5.3	0.063

(%) The number in parenthesis indicates the total proportion of patients with a particular variable. *p* shows the result of the comparison of three groups (*p*-value). ‡ Arithmetic means of 0.5, 1, 2, and 4 kHz. § SRT: speech reception threshold in decibels hearing level. ^+^ SDT: speech discrimination test was at least 80% or greater in all patients and controls. The ability to understand 100% of spoken words from a phonetically balanced list when presented in the left and right ear measured in dB HL.

**Table 3:** Epidemiological and auditory differences between controls and patients with secondary Raynaud Phenomenon (RP).

Variable	Controls	Primary RP	*p*-Value
	(n = 37) (%)	(n = 37) (%)	
Sex (men/women)	2/35	2/35	1
Age at the time of the study (years ± SD)	34.2 ± 6.4	34.5 ± 6.9	0.275
Individuals with abnormal audiovestibular symptoms			
Hearing loss	0 (0)	4 (10.8)	<0.001
Vertigo	0 (0)	4 (11.8)	<0.001
Dizziness	0 (0)	5 (14.7)	<0.001
Disequilibrium	0 (0)	0 (0)	/
Individuals with abnormal hearing loss in the audiogram †	1 (1.7)	4 (10.8)	<0.001
Pure-tone-average (PTA) of air conducted signals in decibels hearing level (dB HL) ‡			
Right ear	8.15 ± 2.30	15.97 ± 1.94	<0.001
Left ear	7.43 ± 1.7	15.67 ± 1.85	<0.001
Pure-tone-average (PTA) of bone-conducted signals in decibels hearing level (dB HL) ‡			
Right ear	8.22 ± 2.43	15.74 ± 1.77	<0.001
Left ear	7.27 ± 1.84	27.19 ± 11.87	<0.001
Absence of stapedial reflex SRT in dB HL §	0 (0)	0 (0)	/
Right ear	9.6 ± 5.4	15.4 ± 7.5	<0.001
Left ear	9.7 ± 5.2	15.7 ± 9.2	<0.002
Abnormal SRT and PTA correlation	/	0 (0)	/
Abnormal SDT ^+^	/	0 (0)	/
SDT in decibels hearing level (dB HL)			
Right ear	23.2 ± 5.6	34.6 ± 11.4	<0.001
Left ear	24.4 ± 4.2	37.7 ± 12.8	<0.001

(%) The number in parenthesis indicates the total proportion of patients with a particular variable. *p*-value shows the result of the comparison of controls vs. the secondary RP group. † Hearing loss was considered present when the audiometric tests disclosed pure-tone thresholds equal to or greater than 30 dB HL of PTA. ‡ Arithmetic means of 0.5, 1, 2, and 4 kHz. § SRT: Speech Reception Threshold in decibels hearing level. ^+^ SDT: The ability to understand 100% of spoken words from a phonetically balanced list when presented in the left and right ear measured in dB HL. The speech Discrimination Test was at least 80% or greater in all patients and controls.

**Table 4:** Hearing thresholds data of pure-tone air and pure-tone bone audiometry test (0.5-1-2-4 kHz) in patients with Primary and Secondary Raynaud Phenomenon (RP) and controls.

Pure-tone air conduction thresholds from the right ear.			
Frequency study (Hz)			*p*-value
500	Control	9.82 ± 0.258	/
	Primary	9.75 ± 0.441	0.996
	Secondary	11.08 ± 0.891	0.169
1000	Control	6.45 ± 0.309	/
	Primary	6.25 ± 0.497	0.989
	Secondary	11.62 ± 1.656	<0.001
2000	Control	6.91 ± 0.693	/
	Primary	6.50 ± 0.734	0.980
	Secondary	16.49 ± 2.316	<0.001
4000	Control	9.45 ± 0.514	0.386
	Primary	6.25 ± 0.497	
	Secondary	23.24 ± 2.755	<0.001
Pure-tone air conduction thresholds from the left ear			
Frequency study (Hz) RP			*p*-value
500	Control	8.27 ± 0.48	/
	Primary	9.25 ± 0.54	0.596
	Secondary	10.0 ± 0.90	0.105
1000	Control	5.64 ± 0.22	/
	Primary	5.0 ± 0.36	0.894
	Secondary	12.03 ± 1.66	<0.001
2000	Control	7.09 ± 0.38	/
	Primary	5.75 ± 0.54	0.792
	Secondary	17.16 ± 2.38	<0.001
4000	Control	8.73 ± 0.45	0.792
	Primary	7.25 ± 0.67	0.813
	Secondary	23.51 ± 2.80	<0.001

Hz (Hertz): unit of frequency in the International System of Units and is defined as one cycle per second, is the audiogram’s frequency measure. *p*-value shows the result of the comparison of pure-tone bone conduction thresholds observed in primary and secondary RP vs. controls.

**Table 5:** Hearing thresholds data of pure-tone bone audiometry test (0.5-1-2-4 kHz) in patients with Primary and Secondary Raynaud Phenomenon (RP) and controls.

Pure-tone air conduction thresholds from the right ear.			
Frequency study (Hz)			*p* value
500	Control	7.27 ± 0.385	----
	Primary	9.00 ± 0.459	0.143
	Secondary	10.81 ± 0.856	<0.001
1000	Control	6.27 ± 0.296	----
	Primary	6.00 ± 0.459	0.979
	Secondary	11.49 ± 1.608	<0.001
2000	Control	7.91 ± 0.658	----
	Primary	6.00 ± 0.459	0.676
	Secondary	16.89 ± 2.254	<0.001
4000	Control	11.45 ± 0.424	----
	Primary	8.75 ± 0.951	0.828
	Secondary	23.78 ± 2.729	<0.001
Pure-tone air conduction thresholds from the left ear			
Frequency study (Hz) RP			*p*-value
500	Control	8.27 ± 0.506	----
	Primary	9.00 ± 0.585	0.77
	Secondary	10.00 ± 0.969	0.13
1000	Control	5.64 ± 0.227	----
	Primary	5.00 ± 0.363	0.875
	Secondary	12.30 ± 1,508	<0.001
2000	Control	6.45 ± 0.382	----
	Primary	6.50 ± 0.639	1
	Secondary	17.97 ± 2.454	<0.001
4000	Control	8.73 ± 0.454	----
	Primary	7.25 ± 0.676	0.828
	Secondary	24.73 ± 2.944	<0.001

Hz: Hertz, unit of frequency in the International System of Units and is defined as one cycle per second, is the audiogram’s frequency measure. *p*-value shows the result of the comparison of pure-tone bone conduction thresholds observed in primary and secondary RP vs. controls.

**Table 6:** Vestibular and postural differences between controls and patients with Primary Raynaud Phenomenon (RP).

Variable	Controls	Primary RP	*p*-Value
	(n = 20) (%)	(n = 20) (%)	
Individuals with abnormal vestibular tests			
Spontaneous nystagmus	0 (0)	0 (0)	/
Evoked nystagmus	0 (0)	0 (0)	/
Abnormal OCR †	0 (0)	0 (0)	/
Patients with positional nystagmus			
With ≥ 1 abnormal position	0 (0)	0 (0)	/
Dix-Hallpike test	0 (0)	0 (0)	/
Abnormal head shaking	0 (0)	0 (0)	/
Abnormal caloric test	0/34 (0)	0/11(0)	/
Abnormal vHIT ‡			
Right gain	0.99 ± 0.06	0.98 ± 0.09	0.792
Left gain	0.99 ± 0.06	0.94 ± 0.08	0.816
Saccades	0 (0)	0 (0)	
Individuals with Abnormal CTSIB §	1/20 (5%)	1/20 (5%)	1
Abnormal CDP	0 (0)	0 (0)	

(%) Number in parenthesis indicates the total proportion of patients with a particular variable. † Abnormal OCR (abnormal oculocephalic response) was considered to be present when the eyes drifted in the same direction as the head and clinically evident compensatory refixation saccades were necessary to reset gaze on the stationary target. vHIT. ‡: video Head Impulse Test. § CTSIB(clinical test of sensory interaction and balance) was considered abnormal when individuals were not able to maintain the position in more than 50% of time. CDP: computerized dynamic posturography. Each of the test items are scored according to the sway, where 100% is no sway and 0% means that the subject falls and then overall postural results were expressed in this way. A composite inferior to 80% was considered abnormal.

**Table 7:** Vestibular and postural differences between controls and patients with Secondary Raynaud Phenomenon (RP).

Variable	Controls	Secondary RP	*p*-Value
	(n = 37) (%)	(n = 37) (%)	
Individuals with abnormal vestibular tests			
Spontaneous nystagmus	0 (0)	0 (0)	/
Evoked nystagmus	0 (0)	0 (0)	/
Abnormal OCR †	0 (0)	0 (0)	/
Patients with positional nystagmus			
With ≥ 1 abnormal position	0 (0)	2 (5.9)	<0.001
Dix-Hallpike test	0 (0)	0 (0)	/
Abnormal head shaking	0 (0)	0 (0)	/
Abnormal caloric test	0/37 (0)	5/27 (18.5)	<0.001
Abnormal vHIT ‡			
Right gain	0.99 ± 0.08	0.91 ± 0.09	0.029
Left gain	0.95 ± 0.07	0.88 ± 0.09	0.013
Saccades	0 (0)	3 (8.8)	0.07
Individuals with Abnormal CTSIB §	1/37(2.7)	9/37(24.3)	<0.001
Patterns of CTSIB visually dependent	1/37(2.7)	1/37(2.7)	/
surface dependent	0/37(0)	2/37(5.4)	/
vestibular loss	0/37(0)	6/37(16.2)	/
Abnormal CDP	0(0)	9/37(24.3%)	<0.001
Patterns of CDP in individuals with abnormal CDP			
visually dependent	0	3	/
vestibular loss	0	5	0.08
somatosensorial selection	0	1	/

(%) Number in parenthesis indicates the total proportion of patients with a particular variable. † Abnormal OCR (abnormal oculocephalic response) was considered to be present when the eyes drifted in the same direction as the head and clinically evident compensatory refixation saccades were necessary to reset gaze on the stationary target. vHIT. ‡: video Head Impulse Test. § CTSIB (clinical test of sensory interaction and balance) was considered abnormal when individuals were not able to maintain the position in more than 50% of time. CDP: computerized dynamic posturography. Each of the test items are scored according to the sway, where 100% is no sway and 0% means that the subject falls and then overall postural results were expressed in this way. A composite inferior to 80% was considered abnormal.

## Data Availability

MDPI Research Data Policies at https://www.mdpi.com/ethics.
